# Distribution and imaging characteristics of spina bifida occulta in young people with low back pain: a retrospective cross-sectional study

**DOI:** 10.1186/s13018-021-02285-w

**Published:** 2021-02-22

**Authors:** Wenhao Li, Zhencheng Xiong, Chunke Dong, Jipeng Song, Liubo Zhang, Jun Zhou, Yanlei Wang, Ping Yi, Feng Yang, Xiangsheng Tang, Mingsheng Tan

**Affiliations:** 1 Beijing University of Chinese Medicine, Beijing, 100029 China; 2 Orthopaedic Department II, China-Japan Friendship Hospital, Beijing, 100029 China; 3 Institute of Medical Technology, Peking University Health Science Center, Beijing, 100191 China; 4 Peking University Third Hospital, Beijing, 100191 China; 5 Beijing Hospital of Traditional Chinese Medicine, Capital Medical University, Beijing, China; 6 Peking Union Medical College, Chinese Academy of Medical Sciences, Beijing, 100730 China

**Keywords:** Spina bifida occulta, Young people, Low back pain, Lamina morphology

## Abstract

**Purpose:**

Spina bifida occulta (SBO) is one of the most common congenital spinal deformities. Although many studies have demonstrated the influence of lumbosacral dysplasia on low back pain (LBP) in young athletes, there have been few studies on SBO among young people in other occupations. The purpose of this study is to investigate the distribution of SBO in young people with LBP and to classify SBO from the perspective of lamina development.

**Methods:**

The X-ray films of 148 young patients with LBP were analyzed to quantify the distribution of SBO and classify abnormal laminae.

**Results:**

Of the 148 patients, 93 (61.49%) had SBO: 83 cases involved S1 alone, 2 involved L5–S1, 5 involved S1–2, 2 involved S1–4, and 1 involved L4–S4. According to the degree of the defect, the patients with SBO were divided on the basis of five grades: 9 patients with grade I, 53 with grade II, 23 with grade III, and 8 with grade IV. The cases were classified by the shape of the laminae into 4 types: 15 cases of type a, 11 cases of type b, 37 cases of type c, and 30 cases of type d.

**Conclusion:**

Among the young people with LBP that we surveyed, SBO is the most common lumbosacral dysplasia, which frequently involves the S1 segment. Most laminae in SBO are in the developmental stage of the spinous process, and an abnormal laminar growth direction and laminar stenosis are the most common laminar morphologies in SBO.

## Introduction

Low back pain (LBP) is the most common musculoskeletal disease [1] and one of the main complaints in spine surgery clinics. Many factors, such as lumbar muscle strain, lumbar disc herniation, and lumbar spondylolisthesis, can cause LBP, which occurs in high-, middle-, and low-income countries and all age groups, from children to the elderly [2]. LBP affects 60 to 85% of the population at least once in the lifetime, with 10 to 20% of the population affected by chronic LBP [3]. Globally, from 1990 to 2015, the number of people who were disabled due to LBP increased by 54%, making LBP the main cause of disability in the world at present. Except for a small number of clear pathological causes, such as fracture, malignant tumor, and infection, the specific cause of most cases of LBP cannot be determined [2].

Spina bifida occulta (SBO), which is one of the most common congenital spinal deformities, is a developmental abnormality common to most young patients with LBP observed in our outpatient department. It is a laminar insufficiency that does not involve the spinal cord and spinal meninges. Although there have been many studies [4–7] that demonstrate the influence of lumbosacral dysplasia and spondylolysis on LBP in young athletes, there have been few studies on the distribution of SBO among young people in other occupations. The above studies investigated the causes of LBP in professional football and basketball players and found that lumbar spondylolysis was the most common underlying cause in this group. However, the intensity of exercise and the load on the lumbar spine of young people in other occupations are less than those of athletes; therefore, their symptoms and abnormal imaging findings deserve our attention and vigilance. So we designed this study to investigate the distribution of SBO in young people with LBP and to classify the laminar morphology of SBO from the perspective of laminar development.

## Materials and methods

### Patients

This is a retrospective cross-sectional study. Through the outpatient information system of the hospital, we retrieved the medical information of all patients who presented with the main complaint of LBP in the orthopedic clinic from January 2019 to June 2019, and obtained a total of 2496 patients. With reference to the World Health Organization’s definition of the age of young people (10–24 years old) [8], considering the actual situation in China, we defined the age range of the included population as 10–25 years old. Based on medical records, blood test results, imaging examination results, and follow-up records, we excluded patients with clear causes such as lower back injury, fracture, tumor, rheumatic immune disease, nervous system disease, urinary system disease, and gynecological disease, and professional athletes. Therefore, 148 patients were finally obtained. Among them, there were 62 males and 86 females, with a median age of 23 years. The minimum age was 13 years. The lumbar X-ray films of the 148 patients were evaluated by three professional senior spine surgeons. And the study was approved by the hospital’s ethics committee.

### Classification according to laminar morphology

**Fig. 1:**
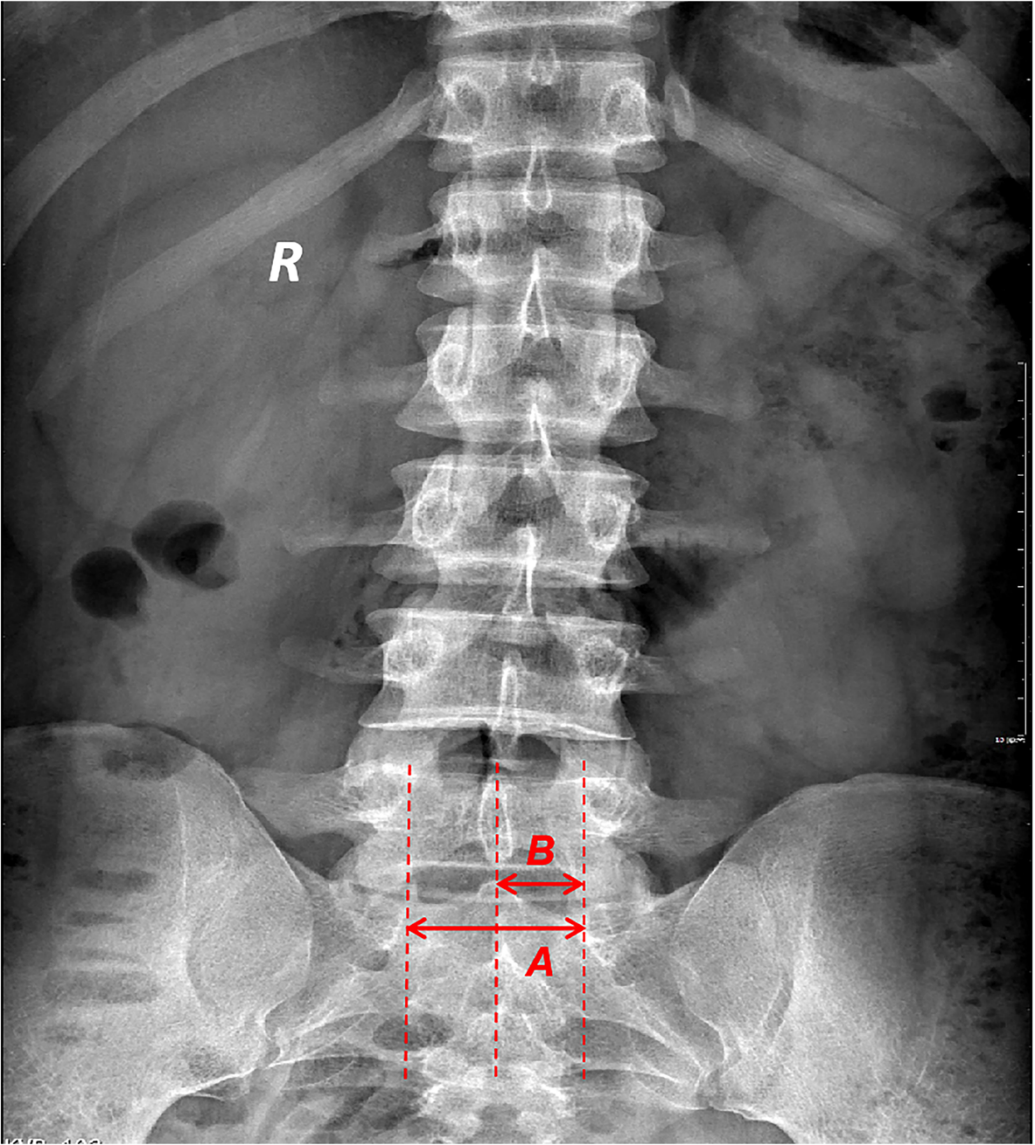
The distance between the inner edges of the pedicle of S1 is transverse diameter A of the spinal canal. The distance from the inner edge of the pedicle to the midline is called the spinal canal radius B, which is half of the transverse diameter of the spinal canal

On a normal lumbar X-ray film, the distance between the inner edges of the pedicle on both sides of the first sacral vertebra (S1) is considered the transverse diameter of the spinal canal, and the distance from the inner edge of the pedicle to the posterior midline is referred to as the spinal canal radius (Fig. 1). The width of the lamina is described as the distance between the upper and lower edges of the lamina, and the direction of the end of the lamina is taken as the direction of laminar development. According to the length of S1 lamina, the degree of defect is divided into five grades: in grade I, the two sides of the lamina are fused into the spinous process; in grade II, the two sides of the lamina are in contact or reach the midline but are not fused into the spinous process; in grade III, the two sides of the lamina are not in contact but both exceed 1/2 of the spinal canal radius; in grade IV, there is no contact between the two sides of the lamina, and one or both sides of the lamina fail to exceed 1/2 of the spinal canal radius; and in grade V, no lamina is seen.

**Fig. 2:**
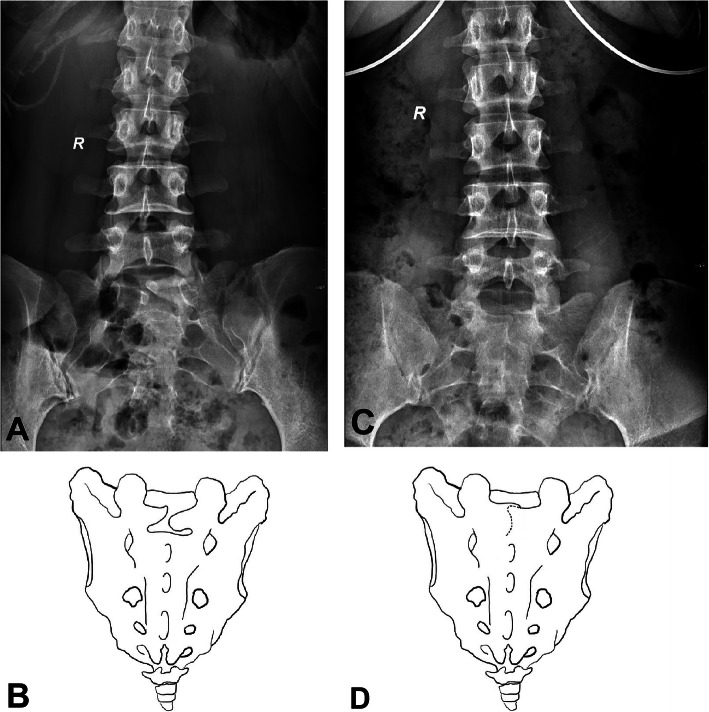
**a** The left lamina grows obliquely upward and the right lamina grows obliquely downward. Although both laminae reach the midline, they cannot meet each other. **b** The schematic diagram. **c** Only the end of the right lamina can be seen, the end of the left lamina is blocked by the right side, and the two lamina overlap, resulting in the inability to meet. **d** The schematic diagram

**Fig. 3:**
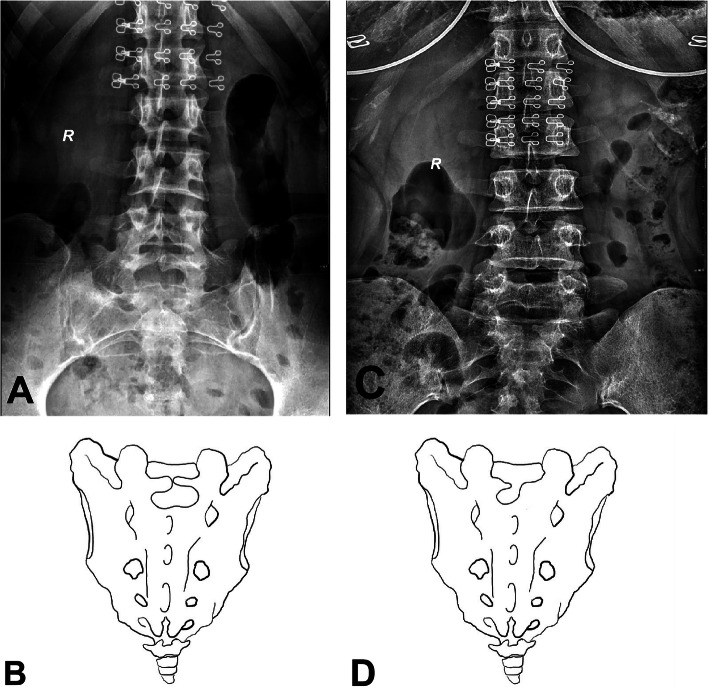
**a** The width of the two sides of the lamina is narrow from the beginning of development, resulting in the failure of fusion with the adjacent lamina below, forming a wide fissure on the surface of the sacrum, as shown in **b**. **c** The left lamina narrows gradually with the development process, as shown in **d**

**Fig. 4:**
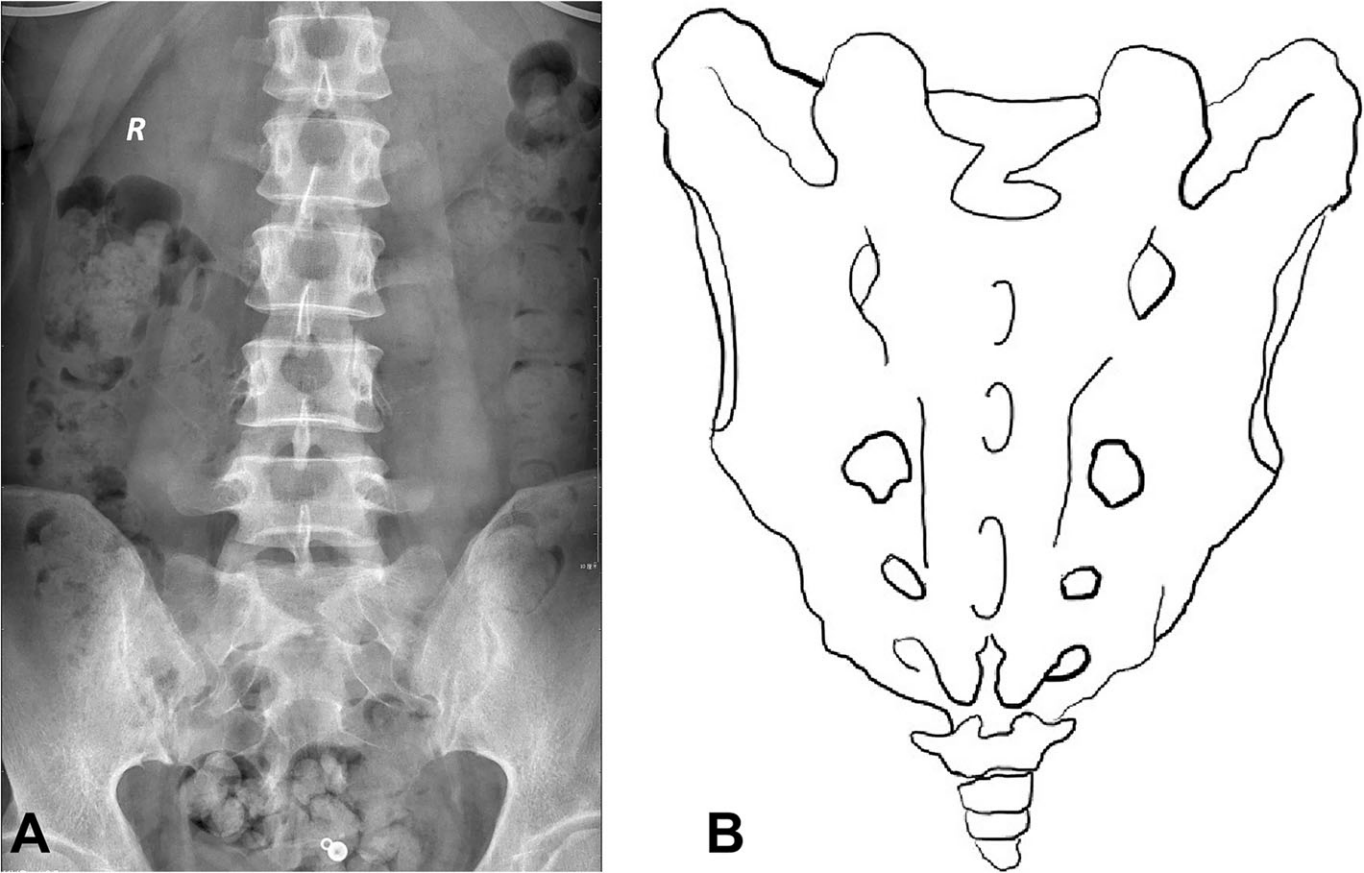
**a** Both an abnormal direction and narrow lamina on both sides of the lamina can be observed, which increases the difficulty of meeting, see diagram **b**

Based on the above five grades, the S1 lamina morphology can be classified into the following categories: type a, normal morphology; type b, abnormal growth direction, in which one side or both sides of the lamina grow above or below the syncline or the two laminae overlap and cannot meet in the midline (Fig. 2); type c, which shows a narrow width, indicating that the lamina is narrow from the beginning of development or gradually narrowed during the development process and resulting in the adjacent lamina below being unable to fuse and the formation of holes or fissures on the surface of the sacrum (Fig. 3); and type d, which is the mixed type in which the lamina has both an abnormal development direction and narrow width (Fig. 4).

## Results

**Fig. 5:**
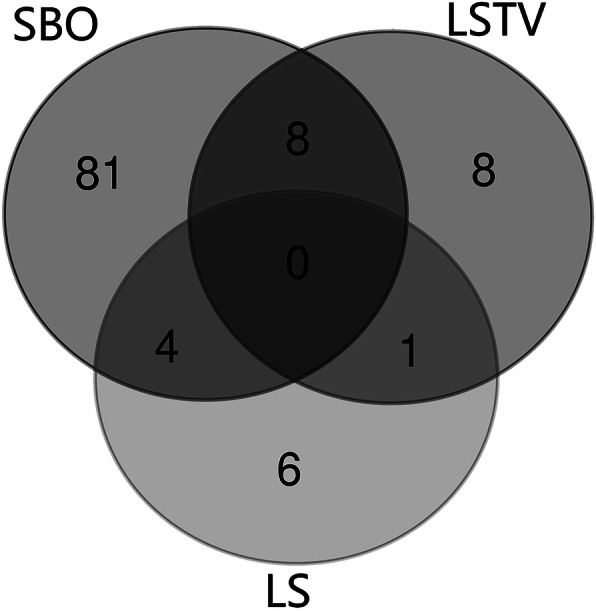
Distribution of various lumbosacral abnormalities

Of the 148 patients, 40 (27.03%) had normal lumbar spines and 108 (72.97%) had abnormal lumbar spine. In patients with abnormal lumbar spine, there were 93 patients (61.49%) with SBO, including 44 males and 49 females. According to the segment, 83 cases occurred in S1 alone, 2 in L5–S1, 5 in S1–2, 2 in S1–4, and 1 in L4–S4. There were 17 cases (11.49%) with lumbosacral transitional vertebrae (LSTV), 8 cases with simple LSTV, 8 cases with SBO, and 1 case with lumbar scoliosis (LS). There were 11 patients (7.43%) with LS, including 6 patients with LS alone and 4 patients with SBO. Some patients exhibited more than one kind of abnormality. The specific distribution is shown in Fig. 5.

**Fig. 6:**
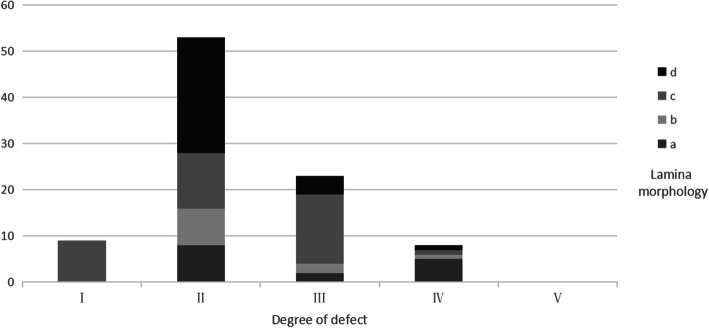
Morphological classification of S1 lamina

In our observation of S1 laminae, we found that 9 patients had grade I defect, all of which were type c in terms of morphology; 53 patients had grade II defect, of which there were 8 patients with type a, 8 patients with type b, 12 patients with type c, and 25 patients with type d morphologies. A total of 23 patients had grade III defect, including 2 patients with type a, 2 patients with type b, 15 patients with type c, and 4 patients with type d morphology; 8 cases had grade IV defect, including 5 cases of type a, 1 case of type b, 1 case of type c, and 1 case of type d. Overall, there were 15 cases of type a, 11 cases of type b, 37 cases of type c, and 30 cases of type d (Table 1 and Fig. 6).

**Table 1:** Morphological classification of S1 lamina

Lamina morphology	Degree of defect					
	I	II	III	IV	V	Total
a	0	8	2	5	0	15
b	0	8	2	1	0	11
c	9	12	15	1	0	37
d	0	25	4	1	0	30
Total	9	53	23	8	0	93

## Discussion

SBO is one of the most common congenital spinal deformities [9] and is usually caused by a failure of laminar fusion without involving the spinal cord and meninges. Approximately 61% of SBO occurs in the lumbosacral region [10]. The prevalence of SBO varies from 1.2 to 50% in different study populations. The largest and most ethnically diverse study to date showed a total prevalence of SBO of 12.4%. Eubanks et al. [11] evaluated 3100 bone specimens and found that a total of 355 cases had SBO, of which 68.7% were white, 31.3% were black, 88.2% were male, and 11.8% were female. Albanese et al. [10] investigated 70 patients and their 222 first-degree relatives and found that among the first-degree relatives, SBO was most common in the siblings and children of patients. Fidas et al. [12] studied the X-rays of 2707 SBO patients and found that SBO was more common in young people, especially in men under 30 years old. The overall prevalence rate of men was twice that of women, and the difference was most obvious under 30 years old.

The prevalence of SBO is dynamic: in some cases, the closure of the vertebral arch may be a continuous process, and the unfused lamina may gradually fuse with the growth and development of bone and the filling of degenerative calcification [9, 11–13], leading to the decline of SBO prevalence with increasing age. This may account for the large differences in the prevalence reported in different studies. Moreover, the prevalence of SBO tends to be increased in younger populations, and the prevalence of SBO in males is increasing [14].

Our study found that 27.03% of young people with LBP had normal lumbar spines. SBO was evident in 61.49% of the patients, exceeding the percentages with LSTV (11.49%) and LS (7.43%). Moreover, SBO could be combined with LSTV and LS. SBO in the single S1 segment alone accounted for 89.25% of the cases, with all of the cases (93 cases) involving the S1 segment, making S1 the most commonly affected segment. Laminae were observed for all SBO cases, with grade II defects being most common, followed by grade III defects. The number of patients with large grade IV and grade V lamina defects was reduced, indicating that most SBO-affected laminae were in the developmental stage of the spinous process. Because our study cohort is relatively young, we cannot rule out the possibility of gradual fusion of the affected laminae with age. There were 9 cases of laminar stenosis in the patients with grade I defects, which indicates that even if the spinous process has been formed, there may be an abnormal lamina morphology. Therefore, spinous process fusion cannot be used as the only index to judge SBO but should also be combined with the assessment of laminar morphology. Regarding the lamina, the meeting point forms the premise for the fusion of the spinous process. Abnormal laminar growth direction and a narrow lamina are the two most common morphological abnormalities, which lead to an inability of the lamina to meet and fuse in the midline. In addition, abnormal laminae accounted for 37.50% of the level IV defect, 91.30% of the level III defect, and 84.90% of the level II defect. It can be seen that with the growth and development of the lamina, the probability of morphological abnormality will increase.

SBO-induced LBP can be explained by local stress concentration, lumbar disc degeneration, muscle attachment area loss, muscle proprioception loss, and additional spinal load. The finite element study have shown that there is a lack of load sharing across the vertebral arch on SBO segments, resulting in stress concentration in the inferior isthmus of the inferior articular process of the lumbar spine and the sacral wing [15, 16]. Some studies have shown that the production of LBP is related to the degeneration of the L4–S1 intervertebral disc [17, 18]. In addition, the loss of the spinous process caused by SBO reduces the attachment area for the multifidus muscle, on the one hand making the patient’s ability to control body position poor and increasing the swing degree [19]; on the other hand, the stability of the lumbar spine is positively related to the proprioception of the muscles, and the multifidus muscle plays an important role in lumbosacral positional sensing [20]. Thus, the loss of attachment for the multifidus muscle will lead to a loss of proprioception [21], which is related to lumbar instability and LBP [22, 23]. In addition, carrying backpacks is a risk factor for LBP in young people. The extra load caused by backpacks and the posture of carrying backpacks may cause considerable spine load. Moreover, the mechanical demand on the lower back caused by the trunk movement caused by backpacks increases, which needs to be balanced by active and passive responses of the lumbar tissue [24].

Our study defined the upper age limit of young people as 25 years old, while the World Health Organization standard is under 24 years old [8]. Because Chinese people have a tradition of using nominal age, which is 1 year older than the actual age, considering this factor, we included patients whose age were registered as 25 years old. And any external factors that can cause LBP, such as trauma, fractures, and tumors, were excluded, and professional athletes were also excluded because their lumbar spine load is much greater than other people. Our research cohort consisted of young people from the outpatient department, whose symptoms and signs were not serious. To avoid excessive medical treatment and extra economic burden, no further three-dimensional CT examinations of the lumbar spine were justified. Thus, the only imaging data available were the lumbar X-rays. We have established morphological rules indicative of abnormal laminae in these X-ray films and classified these abnormal laminae on the basis of previous studies. Fidas et al. [12] classified SBO into four modes with 2 mm as the boundary and whether it was symmetrical in the midline. Based on this, Chang Shin et al. [19] added a new type, the whole sacral involvement type. Solomon et al. [14] classified SBO into 4 categories with 5 mm and 10 mm steps and 5 categories according to the upper and lower positions of the openings. Wu et al. [25] first divided the sacral canal into four grades and then divided SBO into four grades according to the proportion of sacral fissure in the sacral canal. The above scholars mainly classified SBO according to the size and location of the defects to describe the severity of SBO. According to our observations, various abnormal developmental patterns are often the cause of the bilateral laminae failing to meet. Therefore, based on the classifications of the above scholars, we added a classification regarding lamina morphologies, resulting in a classification method that can not only describe the severity of the defects but also the lamina morphologies. According to our analysis, the main reasons for the two sides of the lamina not meeting are as follows: first, the development speed of the lamina is slow; second, the developmental direction of the lamina is abnormal; third, the lamina width is narrow. The length of the lamina can be insufficient due to delayed development of the lamina, and the end of the lamina cannot reach the fusion position. Similar to the abnormal direction of the lamina, the length of the lamina usually changes with the width of the lamina, which shows that the width gradually narrows, the end becomes sharp, and the hole becomes wedge-shaped. We speculate that this may be caused by two abnormal laminar types that seek fusion with the contralateral lamina as much as possible at the expense of width to obtain length. Because the direction of the end of the lamina changes obviously, we take it as the direction of laminar development. The developmental directions of most laminae are relatively intuitive and easy to judge. However, when the two sides of the lamina overlap, the end of one side of the lamina is blocked by the end of the other side of the lamina on the X-ray film, and only one end of the lamina can be seen. Therefore, in the absence of 3D CT, we can also judge the three-dimensional direction of the lamina by this method. On the one hand, the lamina of the sacrum should be fused with the opposite lamina to form the spinous process; on the other hand, it should be fused with the adjacent lamina to form a complete sacral surface. Therefore, when the width of the lamina is narrow, it cannot fuse with the adjacent lamina, and it will develop holes or fissures on the sacral surface. Although most cases of SBO occur in lumbosacral vertebrae, a smaller number of cases also occur in thoracic and cervical vertebrae. When SBO occurs in these places, the classification method described by the scholars mentioned above is no longer applicable, and the degree and shape of the SBO defect in different segments may be different. Our classification method is intended for a certain segment, and has relatively complete observational indicators. Therefore, this classification can be used not only for the S1 segment but also to describe other segments with laminar development abnormalities.

In terms of treatment, the presence of SBO probably has little effect on the choice of treatment plans for patients with LBP, but we believe that how to prevent LBP in patients with SBO is more important than how to treat it. According to our research results, we suggest (1) the routine physical examination of young people should include at least one lumbar spine X-ray examination, which is conducive to early detection of the presence of SBO and the development of appropriate exercise programs for SBO patients; (2) young people who show evidence of SBO upon physical examination, regardless of age and degree of defect, should pay attention to self-protection in work and life, avoid strenuous exercise, reduce the load of lumbar spine, correct bad body posture, and exercise waist muscles. Once symptoms of LBP occur, systematic diagnosis and treatment should be sought as early as possible.

## Limitation

Our study has the following limitations: Compared with other similar studies, the sample size of this study is still not large enough and we lack long-term follow-up data for patients. Since the research object is outpatients, 3D CT examinations of the lumbar spine were not arranged for all patients based on their condition, so our research conclusions lack the support of 3D imaging, which is the direction of our next step. And we have not studied the relationship between the degree of LBP and the degree of laminar defect in more depth, which is also our next research direction.

## Conclusion

In conclusion, among the young people with LBP that we surveyed, SBO is the most common lumbosacral dysplasia, and S1 is the most common segment involved in SBO. In most cases of SBO, the lamina is in the developing stage of the spinous process. An abnormal laminar growth direction and laminar stenosis are the most common morphologies in SBO. The classification of SBO according to the severity of laminar defect helps doctors to more accurately assess the patient’s condition and prognosis, and propose more scientific health guidance.

## Data Availability

The datasets used and/or analyzed during the current study are available from the corresponding author on reasonable request.

## Abbreviations

SBO: Spina bifida occulta
LBP: Low back pain
LSTV: Lumbosacral transitional vertebrae
LS: Lumbar scoliosis
